# A three-gene expression-based risk score can refine the European LeukemiaNet AML classification

**DOI:** 10.1186/s13045-016-0308-8

**Published:** 2016-09-01

**Authors:** Stefan Wilop, Wen-Chien Chou, Edgar Jost, Martina Crysandt, Jens Panse, Ming-Kai Chuang, Tim H. Brümmendorf, Wolfgang Wagner, Hwei-Fang Tien, Behzad Kharabi Masouleh

**Affiliations:** 1 Department of Hematology, Oncology, Hemostaseology and Stem Cell Transplantation, Medical Faculty, RWTH Aachen University, Aachen, Germany; 2 Department of Laboratory Medicine, National Taiwan University Hospital, Taipei, Taiwan; 3 Department of Internal Medicine, National Taiwan University Hospital, Taipei, Taiwan; 4 Helmholtz-Institute for Biomedical Engineering, Stem Cell Biology and Cellular, Engineering, University Hospital of the RWTH Aachen, Aachen, Germany; 5 Institute for Biomedical Engineering - Cell Biology, University Hospital of the RWTH, Aachen, Germany

## Abstract

**Background:**

Risk stratification based on cytogenetics of acute myeloid leukemia (AML) remains imprecise. The introduction of novel genetic and epigenetic markers has helped to close this gap and increased the specificity of risk stratification, although most studies have been conducted in specific AML subpopulations. In order to overcome this limitation, we used a genome-wide approach in multiple AML populations to develop a robust prediction model for AML survival.

**Methods:**

We conducted a genome-wide expression analysis of two data sets from AML patients enrolled into the AMLCG-1999 trial and from the Tumor Cancer Genome Atlas (TCGA) to develop a prognostic score to refine current risk classification and performed a validation on two data sets of the National Taiwan University Hospital (NTUH) and an independent AMLCG cohort.

**Results:**

In our training set, using a stringent multi-step approach, we identified a small three-gene prognostic scoring system, named Tri-AML score (TriAS) which highly correlated with overall survival (OS). Multivariate analysis revealed TriAS to be an independent prognostic factor in all tested training and additional validation sets, even including age, current cytogenetic-based risk stratification, and three other recently developed expression-based scoring models for AML.

**Conclusions:**

The Tri-AML score allows robust and clinically practical risk stratification for the outcome of AML patients. TriAS substantially refined current ELN risk stratification assigning 44.5 % of the patients into a different risk category.

**Electronic supplementary material:**

The online version of this article (doi:10.1186/s13045-016-0308-8) contains supplementary material, which is available to authorized users.

## Key points

- TriAS improves risk stratification in AML
- TriAS is robust in multivariate analysis compared to established risk factors

## Background

The biological heterogeneity of acute myeloid leukemia (AML) in combination with patient-related risk factors such as age or co-morbidities result in a wide range of clinical outcomes making it a continuous challenge for clinicians to assess individual patients’ risk. Currently applied risk-prognostication models mainly rely on a combination of pre-treatment karyotype and molecular mutations. Recent improvements have been made in prognostication, e.g., by adding individual molecular markers to conventional cytogenetics—particularly in patients with normal karyotype AML. The large variability of outcomes within these individual risk groups suggests that more sophisticated approaches including epigenetics [1, 2], microRNA [3], or scoring models based on individual genes [4, 5] are required to provide a more personalized risk assessment. While these studies represent a great leap forward, several of these studies contain certain limitations, often analyzing only a specific AML subset [3, 5], such as cytogenetically normal AML (CN-AML), which only counts for 40 to 50 % of adult and 25 % of pediatric AML patients [6, 7].

In this regard, improved risk stratification is still an unmet clinical need also in elderly AML patients with still poor long-term overall survival (OS) [8]. In order to overcome some of these limitations, we used an unbiased genome-wide approach to identify reliable genetic markers and developed a prognostic scoring system named Tri-AML score (TriAS).

## Methods

### Patients and treatment

In total, four data sets were used in this study. Two independent data sets comprising of total 242 patients served as training sets, including 163 patients from the TCGA portal investigated using RNAseq technology [9] and 79 patients from which 62 were enrolled in the German AML Cooperative Group (AMLCG) 1999 trial [10], while 17 had received therapy outside of the trial [4] using the Affymetrix 133 Plus 2.0 platform (GSE12417-GPL570). Two additional independent validation sets were derived from either 227 patients at the National Taiwan University Hospital (NTUH) [11] (validation set 1) using the Illumina HumanHT-12 v4 Expression BeadChip platform as well as a second set derived from additional 163 patients enrolled in the AMLCG 1999 trial (GSE12417-GPL96A and B, validation set 2) using the Affymetrix 133 Plus 2.0 platform. Clinical characteristics and survival endpoints were used as described in the individual gene expression data sets [4, 9, 11]. Cytogenetic risk groups were available for all data sets, even though the AMLCG data set included CN-AML patients only.

### Identification of prognostic genes

**Fig. 1:**
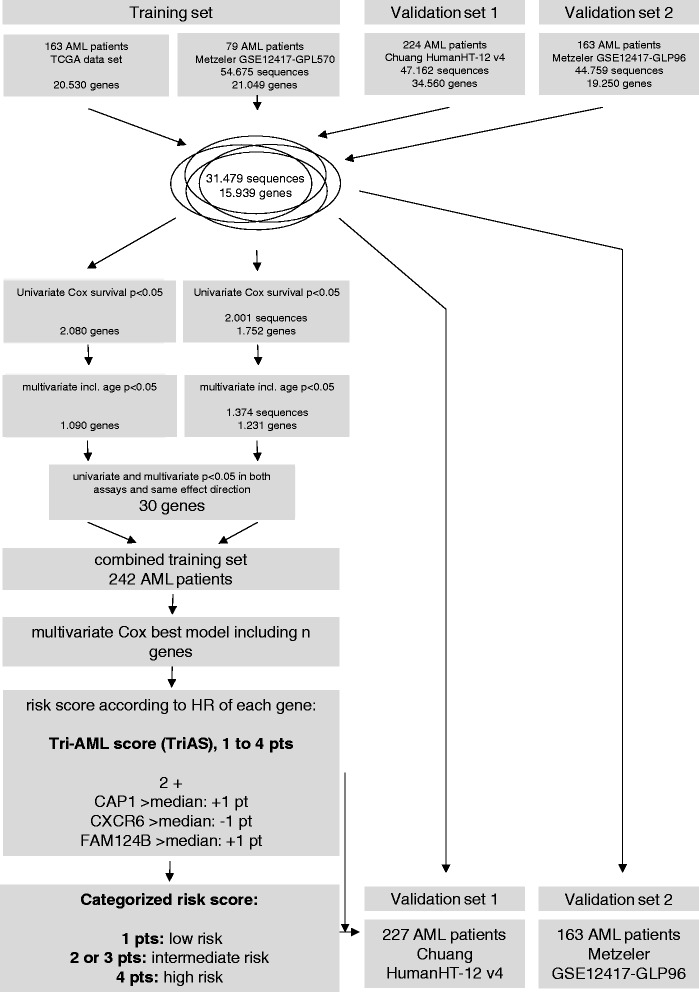
Genome-wide approach to identify a robust prognostic clinical score in AML patients. The schematic overview how to identify a robust AML scoring model using four different expression data sets from TCGA, NTUH, and two independent data sets of the AMLCG-1999 trial (GSE12417-GPL96 and GSE12417-GPL570) is shown. Statistical analysis was conducted either by uni- or multivariate Cox regression analysis

We used a multi-step approach in order to identify the most reliable combination of expression-based markers (Fig. 1). In order to facilitate generation and validation of a score, only transcripts were included in the analysis where the corresponding gene was available in all four data sets. First, univariate Cox regression analysis using the dichotomized expression (higher or lower compared to the median of the corresponding data set) was conducted to identify all genes with significant impact on OS in the training sets (TCGA and GSE570). Next, age was included as a confounding factor into a multivariate Cox regression model of each training subset. Selection of those genes with significant impact on OS in uni- and multivariate analysis including age in both subsets of the training set with the same effect direction and expression of the transcripts in all patient samples led to a candidate list of 30 genes.

### Development of expression-based scoring models

In order to systematically evaluate the prediction ability in the combined training set (TCGA + GSE570) while reducing the number of genes in our model, we calculated each best predicting combination of *n* = 30, *n* = 29… down to *n* = 1 genes using multivariate Cox regression analysis keeping age >65 years and the cytogenetic risk group within the model. The model with the highest likelihood score was identified using the branch-and-bound algorithm of Furnival and Wilson as included in the SAS software package version 9.3.

An individual score was then created for each n-gene-combination: For each of the n-genes included in the score, 1 point was added if expression of the gene was above the median expression of the data set in case of genes with a hazard ratio >1 in the multivariate n-gene-model, whereas 1 point was subtracted in case of a hazard ratio <1 in the multivariate n-gene-model.

### Gene ontology studies

Gene ontology studies were analyzed using the software platform Cytoscape (Version 3.2.1) [12] and the plugin BiNGO (Version 2.4.4) [13]. Testing for significant pathway enrichment in BiNGO was performed using hypergeometric distribution testing and multiple testing correction by Benjamini & Hochberg False Discovery Rate (FDR) with a significance cutoff value of *p* = 0.05.

### Computing previously published AML scores (Marcucci et al., Chuang et al., Li et al.)

For each data set, three recently published expression-based AML scores have been additionally calculated as previously described: the dichotomized Marcucci score [5], the continuous Chuang score[11], and the dichotomized Li score [14].

### Statistical analysis

Gene expression data of each patient was dichotomized based either on a higher (Gene^HI^) or lower (Gene^LOW^) expression value compared to the median of the cohort of the individual data set as a cutoff. For the identification of prognostic genes, univariate Cox regression analysis of overall survival was performed for each single gene using the dichotomized expression of each gene. The median expression for each gene of each microarray data set served as the cutoff between high and low expression. Additionally, multivariate Cox regression was performed including the dichotomized expression and age.

Multivariate Cox regression analysis including several competing risk factors was performed to estimate hazard ratios and 95 % confidence intervals of our scores in different data sets. Categorized scores were additionally plotted using the Kaplan-Meier method. All statistical tests were two-sided and performed using SAS 9.3 (SAS Institute Inc., Cary, NC, USA). For all tests, the level of significance was *p* < 0.05.

## Results

### Using a genome-wide approach to identify robust prognostic markers in AML

In order to identify the most reliable genes for a prediction model, we analyzed 15.939 individual genes and 31.479 sequences available in all four data set (Fig. 1). We found 2.080 genes of the TCGA and 2.001 sequences in 1.752 genes of the GSE12417-GPL570 data set with significant impact on OS using univariate Cox regression analysis. When we included age, one of the strongest predictors of AML survival [15] as a confounding factor, 1.090 genes of the TCGA and 1.374 sequences in 1.231 genes of the GSE570 data set remained significant using multivariate analysis. Of these, 30 genes showed significant impact on OS with the same effect direction in univariate and multivariate analysis in both subsets of the training set (Additional file 1: Table S1).

To assess the functional relevance of these genes, we conducted gene ontology (GO) analysis which revealed significant enrichment in pathways related to cell stress, apoptosis, tyrosine kinase signaling, endocytosis, and cell cycle (Additional file 1: Table S2).

### Computing a prognostic weighted three-gene expression score led to the Tri-AML score

Several studies have already developed large-gene signatures ranging from 7- to 86-gene prognostic signatures or weighted scoring models in AML [3–5, 14]. However, such multi-gene signatures remain difficult to implement into routine clinical application, as we even considered a 30-gene signature less practical for routine use.

To find an appropriately predictive expression signature, while using the lowest number of genes, we first merged both training subsets into one combined training set including 242 patients (Fig. 1). We then calculated all best predicting *n*-out-of-30-gene-scores comprising *n* = 1, up to all of our 30 candidate genes. While the number of possible *n*-out-of-30-gene-scores dramatically increased up to 15 genes included (Additional file 1: Figure S1a), the predictive value of the scores as measured by the multivariate significance level in the combined training set reached a plateau after eight genes (Additional file 1: Figure S1b).

For further routine use, we propose a 3-gene-combination to be most appropriate including a reasonable low number of genes still preserving very high prediction ability. The best predicting 3-gene-score in our training set included the genes, *C-X-C chemokine receptor type 6* (*CXCR6*), *family with sequence similarity 124B* (*FAM124B*), and *adenylyl cyclase-associated protein 1* (*CAP1*) (Fig. 1). While a higher gene expression of *CAP1* and *FAM124B* was associated with adverse survival, a higher expression of *CXCR6* led to a better survival. Taking the hazard ratios of the individual genes in multivariate analysis into account and to end up with positive values only, a score ranging from 1 to 4 points (pts) can be calculated as:

$$ \begin{array}{l}+1\ \mathrm{point},\;\mathrm{if}\ \mathrm{t}\mathrm{he}\ \mathrm{expression}\ \mathrm{o}\mathrm{f}\ \mathrm{CAP}1\ \mathrm{is}>\mathrm{than}\ \mathrm{t}\mathrm{he}\ \mathrm{median}\hfill \\ {}-1\ \mathrm{point},\;\mathrm{if}\ \mathrm{t}\mathrm{he}\ \mathrm{expression}\ \mathrm{o}\mathrm{f}\ \mathrm{CXCR}6\ \mathrm{is}>\mathrm{than}\ \mathrm{t}\mathrm{he}\ \mathrm{median}\hfill \\ {}+1\ \mathrm{point},\;\mathrm{if}\ \mathrm{t}\mathrm{he}\ \mathrm{expression}\ \mathrm{o}\mathrm{f}\ \mathrm{F}\mathrm{A}\mathrm{M}124\mathrm{B}\ \mathrm{is}>\mathrm{than}\ \mathrm{t}\mathrm{he}\ \mathrm{median}\hfill \\ {}+2\operatorname{points}\left(\mathrm{in}\ \mathrm{o}\mathrm{rder}\ \mathrm{t}\mathrm{o}\ \mathrm{end}\ \mathrm{up}\ \mathrm{with}\ \mathrm{positive}\ \mathrm{values}\ \mathrm{o}\mathrm{nly}\right)\hfill \end{array} $$

**Fig. 2:**
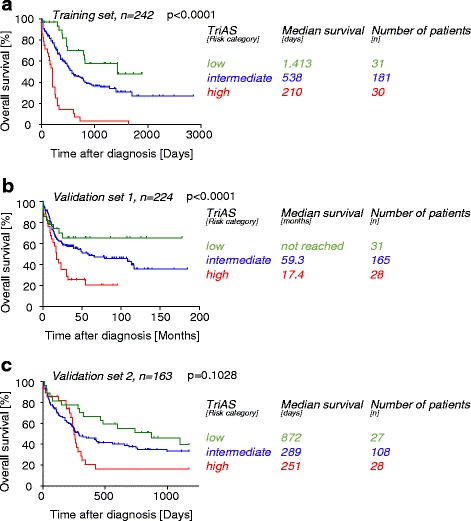
Categorized TriAS predicts overall survival of AML patients. Kaplan-Meier survival analysis of pooled patients from the TCGA data set and enrolled into AMLCG-1999 (GSE12417-GPL570) (training set) (**a**) and two additional validation sets from either NTUH (**b**) or AMLCG-1999 (**c**) based on TriAS categories are shown

This score, named Tri-AML score (TriAS) highly predicted OS in the training set in multivariate Cox analysis including age as a competing risk factor (*p* < 0.0001). To further simplify, TriAS could also be categorized into low-, intermediate-, and high-risk groups allowing reliable segregation (1 pts: low risk, *n* = 31, 2/3 pts: intermediate risk, *n* = 181, 4 pts: high risk *n* = 30, *p* < 0.0001, Fig. 2a). TriAS remained independently significant in multivariate Cox analysis including age in the NTUH validation set 1 (*p* < 0.0001) and showed a trend in the GSE12417-GPL96 validation set 2 (*p* = 0.1028). Kaplan-Meier plots are shown in Fig. 2b, c).

### TriAS predicts survival independently of established risk factors

Current AML risk stratification is based on age and cytogenetic risk group [16]. While the publicly available patient data sets enrolled in the AMLCG-1999 trial only included CN-AML patients with intermediate risk and no further genetic discrimination, this information was readily available in the TCGA and NTUH data sets as previously described [9, 11].

In a direct multivariate analysis of both the TCGA training and the NTUH validation set including cytogenetic risk group and age >65 years, TriAS remained independently predictive for OS in both data sets (Table 1), while clinical parameters were mainly similarly distributed within the different risk groups according to TriAS (Additional file 1: Table S3).

**Table 1:** Multivariate Cox regression analysis for OS including age >65 years, cytogenetic and molecular risk factors, gender, and TriAS in the TCGA training and all validation sets if available are shown

	HR multivariate	*p* value multivariate
TCGA training set (*n* = 154)		
Cytogenetic risk group poor	1.505 (0.896–2.527)	0.1222
Cytogenetic risk group favorable	*0.448 (0.228–0.881)*	*0.0200*
FLT3 mutated	1.590 (0.946–2.672)	0.0799
NPM1 mutated	0.718 (0.419–1.229)	0.2267
Gender (female)	1.192 (0.790–1.797)	0.4033
Age > 65	*4.472 (2.818–7.098)*	*<0.0001*
TriAS	*1.978 (1.505–2.600)*	*<0.0001*
GPL570 training set (*n* = 79)		
Age > 65	1.683 (0.943–3.007)	0.0784
TriAS	*2.147 (1.557–2.961)*	*<0.0001*
NTUH validation set 1 (*n* = 221)		
Cytogenetic risk group poor	*2.504 (1.547–4.052)*	*0.0002*
Cytogenetic risk group favorable	*0.434 (0.234–0.802)*	*0.0077*
CEBPA mutated	*0.248 (0.098–0.629)*	*0.0033*
FLT3 mutated	*2.034 (1.320–3.135)*	*0.0013*
NPM1 mutated	0.801 (0.483*–*1.328)	0.3893
Gender (female)	0.779 (0.526*–*1.152)	0.2109
Age > 65	*2.064 (1.258–3.389)*	*0.0042*
TriAS	*1.393 (1.097–1.769)*	*0.0066*
GPL96 validation set 2 (*n* = 163)		
Age > 65	*1.639 (1.097–2.449)*	*0.0159*
TriAS	*1.239 (1.014–1.515)*	*0.0361*

italic *p*-values relate to significant findings (*p*<0.05)

In particular, since the older patients (age > 65 years) remain a difficult-to-treat subpopulation, we then applied TriAS to this age population using the combined four data sets (*n* = 632). Even within the older patient cohort (*n* = 166), TriAS also allowed clear segregation of different risk groups (median OS TriAS 1: 26.8 months, TriAS 2/3: 8.3 months, TriAS 4: 4.1 months; *p* < 0.0001).

Similarly, TriAS was also able to predict relapse-free survival (RFS) in direct multivariate analysis including age, gender, and molecular and cytogenetic risk factors, only available in the NTUH validation set 1 (*p* = 0.03; Additional file 1: Table S4).

### TriAS predicts independently of previously identified gene expression scores

Recently, three highly predictive scoring models were developed, such as a 7-gene score by Marcucci et al. [5] and an 11-gene expression score both developed for CN-AML patients proposed by Chuang et al. [11] or a 24-gene score by Li et al. [14] including several cytogenetically defined subsets. All three models were able to predict survival in our applied data sets in univariate analysis (data not shown). Notably, including cytogenetic risk, gender, and age >65 as well as all four expression-based risk scores into one multivariate Cox regression model, only age >65 years and TriAS remained independently predictive in the TCGA training set as well as the NTUH validation set (Table 2).

**Table 2:** TriAS independently segregates survival of AML patients even including other expression-based risk scores: multivariate Cox regression analysis for OS of AML patients from the TCGA training and the NTUH validation set 1 using cytogenetic risk, gender, age >65 years, and TriAS as well as scores developed by Marcucci, Chuang, and Li

	HR multivariate	*p* value multivariate
TCGA training set (*n* = 161)		
Cytogenetic risk group poor	1.516 (0.887*–*2.594)	0.1284
Cytogenetic risk group favorable	0.651 (0.329*–*1.290)	0.2186
Gender (female)	1.343 (0.894*–*2.018)	0.1557
Age > 65	*3.817 (2.429–5.999)*	*<0.0001*
Marcucci score	1.090 (0.661*–*1.800)	0.7349
Chuang score	0.993 (0.949*–*1.039)	0.7603
Li score	1.432 (0.861*–*2.380)	0.1661
TriAS	*2.148 (1.659–2.782)*	*<0.0001*
NTUH validation set (*n* = 221)		
Cytogenetic risk group poor	*2.229 (1.424–3.489)*	*0.0005*
Cytogenetic risk group favorable	*0.473 (0.256–0.873)*	*0.0167*
Gender (female)	0.856 (0.583–1.257)	0.4283
Age > 65	*2.252 (1.378–3.680)*	*0.0012*
Marcucci score	0.952 (0.591–1.533)	0.8402
Chuang score	*1.081 (1.040–1.124)*	*<0.0001*
Li score	1.126 (0.710–1.786)	0.6133
TriAS	*1.337 (1.031–1.733)*	*0.0285*

italic *p*-values relate to significant findings (*p*<0.05)

Since all four scoring models have been developed in different training data sets, we then evaluated the prediction ability in all data sets. While the score proposed by Li et al. included patients with favorable and adverse cytogenetics, both scores proposed by Chuang et al. and Marcucci et al. were developed for CN-AML only. We nevertheless tested their fitness in both the CN-AML and non-CN AML subcohorts: Including age >65 and gender as competing risk factors, only TriAS remained independently significant in the CN patients of the TCGA training set, and TriAS and the Chuang score remained significant in the CN patients of the NTUH validation set 1 (Additional file 1: Table S5).

However, in the non-CN patients of the TCGA training set, only TriAS remained significant, whereas in the subcohort of non-CN patients of the NTUH validation set 1, all four expression-based scoring models were able to significantly predict survival independently of age >65, gender, and the cytogenetic risk group (Additional file 1: Table S6).

In order to test if expression-based scoring models could be used synergistically, we sequentially combined TriAS with each of the other three scores. Comparing “double low risk” (TriAS = 1 and additional other low risk score), “double high risk” (TriAS = 4 and additional other high risk score), and “remaining” patients, each combination (TriAS/Marcucci, TriAS/Chuang, TriAS/Li) also allowed significant segregation in both the training and validation set 1 (Additional file 1: Figure S2a-f). Moreover, comparison of “quadruple low risk,” “quadruple high risk,” and “remaining” patients utilizing all four scores also identified subgroups with significant difference in OS (Additional file 1: Figure S3a-c). But as expected, the addition of several scores only partially improved the prognostic segregation ability.

### TriAS can further refine the European LeukemiaNet AML classification

**Fig. 3:**
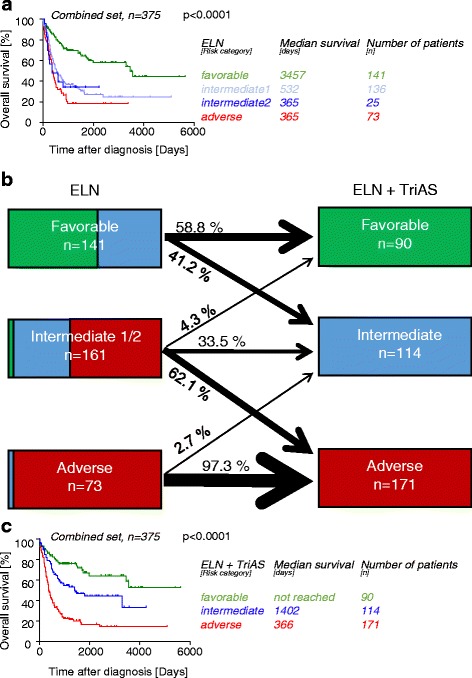
TriAS refines the ELN classification to better segregate AML survival. Overall survival of the AML patients from the TCGA and NUTH data sets according to current ELN classification is shown (**a**) or after implementation of the ELN and TriAS classification (ELN + TriAS) (**c**). The fraction of patients reclassified based on original ELN and ELN + TriAS risk classification is shown (**b**)

Current risk stratification of AML patients is mainly based on the European LeukemiaNet (ELN) classification [17]. Patients are classified into four risk groups (favorable, intermediate 1/2, and adverse) according to cytogenetics and molecular profile based on mutational status of C/EBPα, NPM1, and FLT3. Applying the ELN classification in the combined data sets with cytogenetic and molecular data allowed robust segregation of OS (Fig. 3a).

In each ELN risk group, high TriAS identified a substantial number of patients with shorter survival allowing a more refined definition of patient’s risk (Fig. 3b). Based on median as well as 3-year OS, we developed a combined ELN + TriAS risk stratification which allowed improved risk segregation (Table 3; Fig. 3c). Overall, 167 (44.5 %) of 375 patients in our data sets with ELN risk available were classified into a different risk category if TriAS was included.

**Table 3:** Combination of current ELN risk classification with TriAS leads to a refined ELN + TriAS classification showing three groups with adverse (≤25 %), intermediate (50–60 %), and favorable (>60 %) survival after 3 years

ELN risk	TriAS	Number of patients	3-year OS (%)	Median OS (days)	Refined ELN + TriAS
Favorable	1	24	77.4	Not reached	Favorable
Favorable	2	59	75.4	3555	Favorable
Intermediate 1/2	1	7	68.6	Not reached	Favorable
Favorable	3	46	58.1	1805	Intermediate
Favorable	4	12	52.1	Not reached	Intermediate
Intermediate 1/2	2	54	50.9	1193	Intermediate
Adverse	1	2	50.0	Not reached	Intermediate
Intermediate 1/2	3	78	25.6	366	Adverse
Intermediate 1/2	4	22	23.9	214	Adverse
Adverse	2	24	21.4	368	Adverse
Adverse	3	30	18.2	347	Adverse
Adverse	4	17	0.0	214	Adverse

## Discussion

In this study, we used genome-wide expression and clinical data of multiple independent patient cohorts to propose a simple three-gene expression-based scoring system named TriAS. Although a variety of different risk factors have been described in AML including patient-based factors, genetic and epigenetic changes, and response to therapy [18], age and karyotype remain the most important factors currently used in clinical routine. TriAS showed to be a reliable independent predictor of OS and RFS even in combination with established risk factors and previously published scores in our training sets as well in our validation sets.

The ELN recommendation is a commonly used risk classification scheme for adult AML patients [17]. While ELN risk classification has substantially improved our understanding how to identify high-risk patients with poor overall survival, a subset of these patients is not properly identified by this approach. Therefore, there have been several approaches to further redefine patients at higher risk. As such, a benchmark score proposed by Li et al. similarly redefined poor (adverse) patients using their 24-gene score. This approach allowed to expand the identified high-risk cohort from 27 % (ELN alone) to 52 % (ELN + 24 gene score). Applying TriAS, we were able to distinguish the individual patients’ risk in more detail. Only 58.8 % of prior ELN favorable patients remained in the favorable cohort when TriAS was applied. Subsequently, the ELN intermediate risk groups (1 and 2) can be further segregated between a favorable, intermediate, and adverse risk profile. In particular, within this group, 62.1 % were actually of adverse risk. This is also supported by the findings by Li et al., who showed that a substantial number of patients are of significant higher clinical risk with a substantially shorter overall survival. Applying TriAS could help to identify patients with ELN favorable or intermediate prognosis who nonetheless may benefit from intensified treatment regimens.

For prediction of OS in AML patients, several other studies used a genome-wide approach. An 86-gene signature for CN-AML patients was described by Metzeler et al., which despite its predictive value remains difficult to implement due to the large gene number and has also not been evaluated in non-CN AML subsets so far [4]. In comparison, a more recent study used both genetic and also epigenetic information to predict survival of AML. While this elegant study by Marcucci et al. showed a highly attractive approach to improve risk stratification, again this study was only conducted in the CN-AML subset [5]. Additionally, an 11-gene score for CN-AML patients was developed by Chuang et al. [11] and a 24-gene score by Li et al. for both CN and non-CN AML patients. The score developed by Li et al. allowed an excellent segregation also in combination with established cytogenetic risk stratification [14]. The combined use of the Li and TriAS score led to a further discrimination of OS, notably using only three additional genes. Moreover, the scores did not markedly overlap as combining multiple expression-based risk scores allowed further segregation of patient cohorts. This supports the notion that several gene expression-based prediction scores can be used synergistically and opens an important novel avenue how scoring models could be used in clinical application.

However, our calculations of multiple *n*-out-of-30 scores clearly demonstrated that including a larger number of genes into a scoring model holds an advantage for its predictive value, but this improvement is saturated after a limited number of genes. Since most algorithms include the strongest predictors into a model first, the absolute improvement naturally decreases with each additionally predictor added. In our model, inclusion of more than eight genes did not lead to any relevant further improvement. However, weighing the number of genes to be analyzed in clinical practice and the ability of prediction, a 3-gene-score remained most reasonable in this respect.

Functionally, the three most prognostic genes are only partially described. CXCLR6 has shown to be the cognate receptor of its natural ligand CXCL16 and was initially described on peripheral blood leukocytes and to be present in the bone marrow and prostate [19, 20]. CXCR6/CXCL16 has also been described as an oncogenic axis in a variety of solid cancers, such as papillary thyroid carcinoma, gastric, prostate, and breast cancer, through positive regulation of survival pathways such as ERK [21–25]. Surprisingly, in our data sets, a high expression of CXCR6 led to an improved survival of AML patients. Therefore, subsequent functional studies have to show if this axis might have cancer-type specific functions suggesting to act either oncogenic or tumor suppressive.

The second gene in our scoring model, *FAM124B*, has just recently been identified, and its function remains to be still fully understood. So far, FAM124B has been shown to be an interaction partner for the chromodomain helicase DNA binding protein 7 and 8 genes (CHD7 and CHD8). Mutations of *CHD7* are the major cause for the CHARGE syndrome [26]. On note, it has to be mentioned that so far, there are no studies showing a direct link between the CHARGE syndrome and the onset of leukemia, except one case study describing the co-existence of myelodysplastic syndrome (MDS) [27] and the CHARGE syndrome in an infant. For the interaction partner of FAM124B, CHD8, one study so far described an oncogenic role in a mouse model for BCR-ABL1^+^ acute lymphoblastic leukemia [28], while neither the role of FAM124B nor its interaction with CHD7/8 have been described so far in AML.

The third identified gene, *CAP1*, is an actin-regulating protein which has been shown to promote tumor growth and migration of solid cancers such as HCC, glioma, or breast cancer [29–32]. These oncogenic functions are mediated also by the ERK pathway as shown in breast cancer cells [33]. While no direct oncogenic role for *CAP1* was so far described in AML, one study [34] elucidated its role as direct interacting partner for the insulin resistance protein resistin which is secreted by monocytes [35]. Relevant to mention is that the model which was used to study this interaction was based on the human monocytic THP-1 cell line, which was derived from an AML patient [36]. While these findings indicate a potential role for CAP1 in AML, direct functional evidence so far is missing. In contrast to CXCR6, high expression of CAP1 and/or FAM124B led to impaired overall survival in our data sets in accordance to a suspected oncogenic role of these two genes.

## Conclusions

In summary, our 3-gene expression-based score TriAS allowed robust prediction of AML survival independently of previously identified risk factors with a reasonable low number of genes to be analyzed in clinical practice. The addition of TriAS to the current ELN risk classification allowed a refined risk classification and might help to identify patients who may benefit from intensified treatment. However, future research is required to validate the robustness of the score prospectively in clinical trials. So far, all published scoring models including our own rely on microarray data. With our simple 3-gene-score, future routine clinical application may come within reach if our results can be confirmed using harmonized quantitative real-time PCR.

## Additional file

Additional file 1:
**Table S1**. Statistical analysis and hazard ratios of the final significant 30 genes. **Table S2**. Predictive AML genes are related to cellular stress and apopotosis pathways. **Table S3**. The clinical characteristic based on the categorized TriAS risk groups. **Table S4**. TriAS independently predicts RFS of AML patients independently of other established risk factors. **Table S5**. Multivariate Cox regression analysis for OS of the CN-AML in different prediction scores. **Table S6**. Multivariate Cox regression analysis for OS of the non-CN-AML in different prediction scores. **Figure S1**. The significance level based on incremental number of genes used. **Figure S2**. Combination of risk scores improves the segregation of overall survival of AML patients. **Figure S3**. Four risk scores can be used synergistically to segregate overall survival of AML patients. (PDF 360 kb)

## Abbreviations

AML: Acute myeloid leukemia
TCGA: Tumor Cancer Genome Atlas
NTUH: National Taiwan University Hospital
TriAS: Tri-AML score
CN-AML: Cytogenetically normal AML
FDR: False discovery rate
GO: Gene ontology
RFS: Relapse-free survival
pts: Points
OS: Overall survival
ELN: European LeukemiaNet
HCC: Hepatocellular carcinoma
ALL: Acute lymphoblastic leukemia
