# Supplementary material for: A three-gene expression-based risk score can refine the European LeukemiaNet AML classification
Source: J Hematol Oncol. 2016 Sep 1;9(1):78. doi: 10.1186/s13045-016-0308-8 (PMC5009640; doi:10.1186/s13045-016-0308-8)
Supplement: Additional file 1: — Table S1. Statistical analysis and hazard ratios of the final significant 30 genes. Table S2. Predictive AML genes are related to cellular stress and apopotosis pathways. Table S3. The clinical characteristic based on the categorized TriAS risk groups. Table S4. TriAS independently predicts RFS of AML patients independently of other established risk factors. Table S5. Multivariate Cox regression analysis for OS of the CN-AML in different prediction scores. Table S6. Multivariate Cox regression analysis for OS of the non-CN-AML in different prediction scores. Figure S1. The significance level based on incremental number of genes used. Figure S2. Combination of risk scores improves the segregation of overall survival of AML patients. Figure S3. Four risk scores can be used synergistically to segregate overall survival of AML patients. (PDF 360 kb) [file 13045_2016_308_MOESM1_ESM.pdf]

Supplemental File **May 2016**

A three-gene expression based risk score can refine the European LeukemiaNet AML classification

Stefan Wilop[1], Wen-Chien Chou[2,3], Edgar Jost[1], Martina Crysandt[1], Jens Panse[1], Ming-Kai Chuang[2], Tim H. Brümmendorf[1], Wolfgang Wagner[4,5], Hwei-Fang Tien[3] & Behzad Kharabi Masouleh[1]

- **Supplemental Figures 1 - 3**
- **Supplemental Tables 1 - 6**

Supplemental Figure 1: The significance level improves with incremental number of genes

a

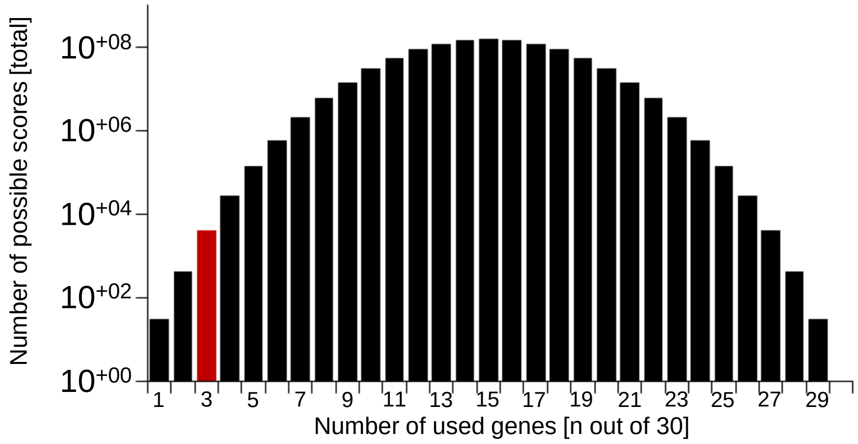

b

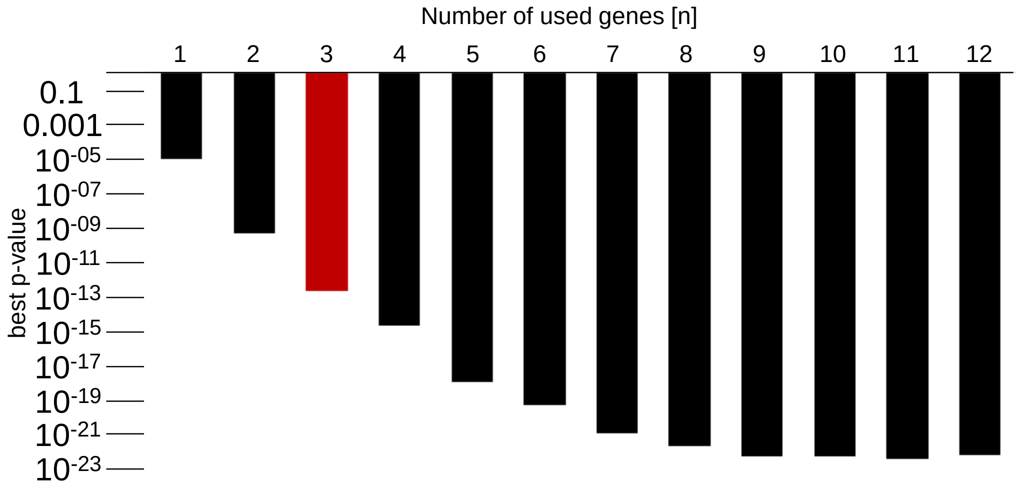

Supplemental Figure 1: The significance level improves with incremental number of genes

Each given number of candidate genes allows the creation of a large number of possible scores. The number of possible n-out-of-30 combinations **(a)** and the lowest multivariate p-value of the best n-out-of -30 score in a multivariate model including age and cytogenetic risk group in the training set **(b)** are shown.

Supplemental Figure 2: Combination of risk scores improves the segregation of overall survival of AML patients

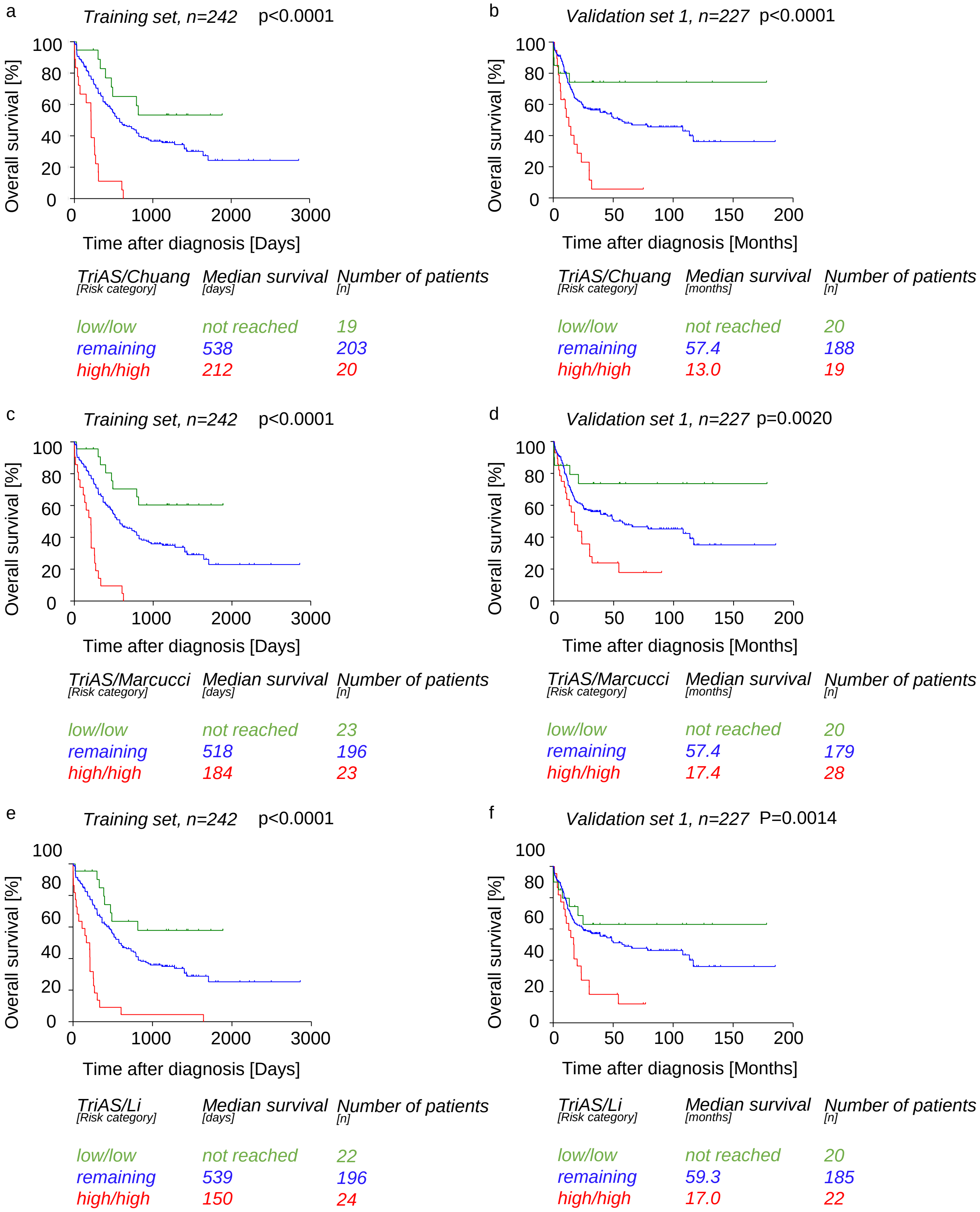

Supplemental Figure 2: Combination of risk scores improves the segregation of overall survival of AML patients

Expression based risk scores can be used synergistically. The OS according to the combination of either the TriAS/Chuang **(a, b)**, TriAS/Marcucci **(c, d)** as well as the TriAS/Li scores **(e, f)** for the training set **(a, c, e)** as well as for the NTUH validation set 1 **(b, d, f)** are shown.

Supplemental Figure 3: Four risk scores can be used synergistically to segregate overall survival of AML patients

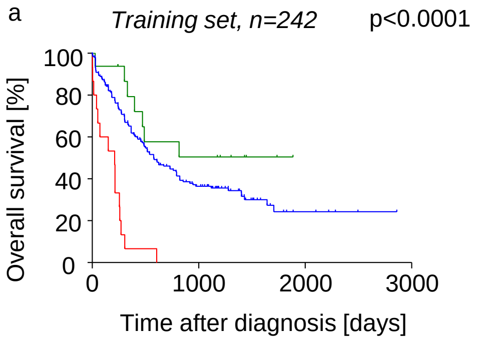

TriAS/Chuang/Marcucci/Li [Risk category]	Median survival [days]	Number of patients [n]
low/low/low/low	Not reached	16
remaining	539	209
high/high/high/high	150	17

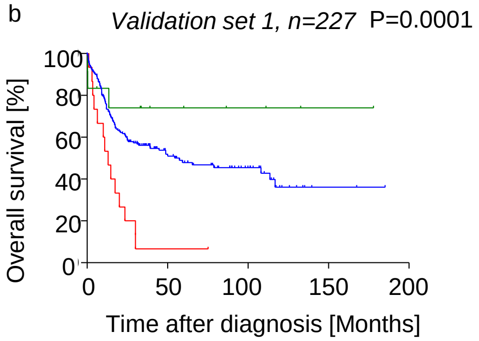

TriAS/Chuang/Marcucci/Li [Risk category]	Median survival [months]	Number of patients [n]
low/low/low/low	Not reached	15
remaining	57.4	200
high/high/high/high	13	12

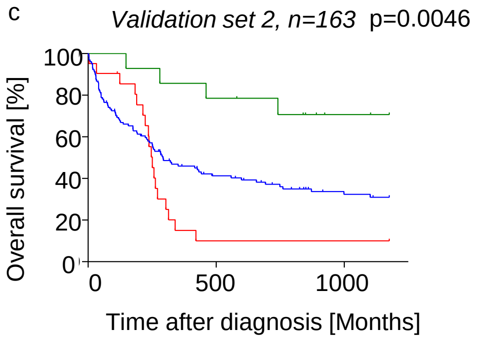

TriAS/Chuang/Marcucci/Li [Risk category]	Median survival [dayss]	Number of patients [n]
low/low/low/low	Not reached	14
remaining	293	128
high/high/high/high	251	21

Supplemental Figure 3: Four risk scores can be used synergistically to segregate overall survival of AML patients

Expression based risk scores can be used synergistically. The OS according to the combination of all four expression based scores (TriAS, Chuang, Marcucci, Li) for the training set **(a)** as well as for the validation set 1 **(b)** and set 2 **(c)** are shown.

Supplemental Table 1: Statistical analysis and hazard ratios of 30 genes using univariate as well as multivariate Cox regression analysis including age as a confounding factor in patients from the TCGA and enrolled in the AMLCG-1999 trial (GSE12417-GPL570) are shown.

Gene name	HR uni [TCGA]	P-value uni [TCGA]	HR multi [TCGA]	P-value multi [TCGA]	HR uni [GSE570]	P-value uni [GSE570]	HR multi [GSE570]	P-value multi [GSE570]
APPL2	0.656	0.0326	0.646	0.0264	0.537	0.0367	0.528	0.032
ATP9B	0.582	0.0062	0.615	0.0146	0.429	0.0054	0.47	0.0137
CAP1	1.976	0.0006	1.764	0.0049	2.081	0.014	2.324	0.0053
CCDC137	1.712	0.0065	1.655	0.0114	2.116	0.0119	1.858	0.0412
CD97	1.867	0.0017	1.672	0.0102	2.362	0.0041	2.251	0.0068
CDYL	0.668	0.0409	0.638	0.0233	0.532	0.0337	0.496	0.0195
CPT1A	1.511	0.0366	1.517	0.0348	2.031	0.0186	1.962	0.0251
CXCR6	0.671	0.0423	0.465	0.0002	0.419	0.0042	0.445	0.0083
DCBLD2	0.676	0.047	0.585	0.0074	0.546	0.0423	0.52	0.0292
DNM1	0.572	0.0052	0.628	0.0212	0.424	0.0052	0.473	0.015
EPAS1	0.592	0.0083	0.552	0.0029	0.532	0.033	0.558	0.0493
FAM124B	1.685	0.0084	1.531	0.0312	3.071	0.0003	2.871	0.0006
FHL1	1.791	0.0034	1.488	0.0497	2.177	0.0097	2.098	0.0141
FRYL	0.656	0.0322	0.616	0.0148	0.381	0.0017	0.366	0.0011
IGF2BP3	2.147	0.0002	1.669	0.0146	1.926	0.028	1.978	0.0232
LEF1	0.67	0.0416	0.628	0.0189	0.37	0.0013	0.432	0.0079
LRRN3	0.561	0.0033	0.631	0.0215	0.477	0.0141	0.516	0.0286
LY9	0.666	0.0388	0.649	0.0286	0.436	0.0061	0.523	0.0379
MYH11	0.561	0.0036	0.629	0.0212	0.494	0.019	0.528	0.0348
OXCT1	1.475	0.0479	1.537	0.03	1.85	0.0388	1.892	0.0328
PBX2	0.648	0.0281	0.586	0.0073	0.468	0.0125	0.514	0.0294
PSMA7	1.964	0.0007	1.79	0.0037	1.883	0.032	2.084	0.0137
RECK	1.778	0.0038	1.582	0.0216	2.344	0.0046	2.38	0.004
RPS6KA1	2.074	0.0003	2.217	0.000099	1.847	0.0381	1.793	0.0492
SLC14A1	0.592	0.009	0.664	0.0412	0.529	0.0312	0.49	0.0164
SORT1	1.794	0.0032	1.593	0.0195	1.875	0.0342	1.96	0.0242
SPDYA	0.614	0.0131	0.622	0.0163	0.517	0.0272	0.484	0.016
TBL1XR1	0.649	0.0275	0.599	0.0101	0.474	0.0129	0.44	0.0065
TRAT1	0.587	0.0073	0.565	0.004	0.363	0.001	0.317	0.0002
ZBTB4	0.609	0.013	0.571	0.0053	0.519	0.0299	0.544	0.0442

Supplemental Table 2: Predictive AML genes are related to cellular stress and apopotosis pathways. Gene ontology classes of the significant genes using BiNGO plugin of the Cytoscape software are shown.

GO ID	Gene ontology class description	p-value	Corrected p-value
43620	regulation of transcription in response to stress	4.70E-05	2.29E-03
7169	transmembrane receptor protein tyrosine kinase signaling pathway	1.13E-04	3.71E-03
6950	response to stress	3.76E-04	6.95E-03
31325	positive regulation of cellular metabolic process	1.47E-03	1.96E-02
43154	negative regulation of caspase activity	1.66E-03	2.11E-02
10972	negative regulation of G2/M transition of mitotic cell cycle	2.20E-03	2.35E-02
6897	endocytosis	2.84E-03	2.80E-02
34142	toll-like receptor 4 signaling pathway	2.91E-03	2.80E-02
45893	positive regulation of transcription. DNA-dependent	3.87E-03	3.12E-02
60070	canonical Wnt receptor signaling pathway	4.17E-03	3.34E-02
45941	positive regulation of transcription	4.24E-03	3.37E-02
16573	histone acetylation	4.62E-03	3.42E-02
42981	regulation of apoptosis	6.19E-03	3.87E-02
43067	regulation of programmed cell death	6.35E-03	3.87E-02
82	G1/S transition of mitotic cell cycle	7.24E-03	3.87E-02
1817	regulation of cytokine production	7.29E-03	3.87E-02
10941	regulation of cell death	7.68E-03	4.00E-02
30099	myeloid cell differentiation	8.31E-03	4.12E-02
43555	regulation of translation in response to stress	1.10E-02	4.67E-02
43281	regulation of caspase activity	1.18E-02	4.93E-02

Supplemental Table 3: The clinical characteristics as available from the TCGA and NTUH data sets of the individual patient cohorts based on the categorized TriAS risk groups are shown.

TCGA Training-Set [n=163] Parameter	Total	TriAS low	TriAS intermediate	TriAS high	p-value (ChiSq)
**Age**					0.7283
≤ 65 years	114	12 (75.00%)	94 (70.15%)	8 (61.54%)	
> 65 years	49	4 (25.00%)	40 (29.85%)	5 (38.46%)	
**Gender**					0.5466
Male	88	7 (43.75%)	75 (55.97%)	6 (46.15%)	
Female	75	9 (56.25%)	59 (44.03%)	7 (53.85%)	
**Cytogenetic risk**					**0.0481**
Favorable	32	7 (43.75%)	25 (18.80%)	0 (0.00%)	
Intermediate	97	6 (37.50%)	81 (60.90%)	10 (83.33%)	
Adverse	32	3 (18.75%)	27 (20.30%)	2 (16.67%)	
**Karyotype**					0.0749
Aberrant	66	10 (62.50%)	54 (44.63%)	2 (18.18%)	
Normal	82	6 (37.50%)	67 (55.37%)	9 (81.82%)	
Unknown	15				
**FLT3-ITD**					**0.0250**
unmutated	110	10 (66.67%)	95 (74.22%)	5 (38.46%)	
mutated	46	5 (33.33%)	33 (25.78%)	8 (61.54%)	
Unknown	7				
**NPM1**					**0.0342**
unmutated	118	14 (87.50%)	98 (74.81%)	6 (46.15%)	
mutated	42	2 (12.50%)	33 (25.19%)	7 (53.85%)	
Unknown	3				

NTUH Validation-Set [n=227] Parameter	Total	TriAS low	TriASs intermediate	TriAS high	p-value
**Age**					0.2108
≤ 65 years	192	21 (75.00%)	140 (84.85%)	31 (91.18%)	
> 65 years	35	7 (25.00%)	25 (15.15%)	3 (8.82%)	
**Gender**					0.3922
Male	118	15 (53.57%)	89 (53.94%)	14 (41.18%)	
Female	109	13 (46.43%)	76 (46.06%)	20 (58.82%)	
**FAB type**					**0.0015**
0	2	0 (0.00%)	2 (1.21%)	0 (0.00%)	
1	55	7 (25.00%)	44 (26.67%)	4 (11.76%)	
2	73	4 (14.29%)	52 (31.52%)	17 (50.00%)	
3	26	10 (35.71%)	15 (9.09%)	1 (2.94%)	
4	55	4 (14.29%)	41 (24.85%)	10 (29.41%)	
5	12	3 (10.71%)	7 (4.24%)	2 (5.88%)	
6	4	0 (0.00%)	4 (2.42%)	0 (0.00)	

**Cytogenetic risk**					0.0974
Favorable	52	10 (40.00%)	34 (20.86%)	8 (24.24%)	
Intermediate	121	12 (48.00%)	96 (58.90%)	13 (39.39%)	
Adverse	41	2 (8.00%)	29 (17.79%)	10 (30.30%)	
Unknown	13				
**Karyotype**					0.0671
Normal	110	14 (56.00%)	74 (45.40%)	22 (66.67%)	
Aberrant	111	11 (44.00%)	89 (54.60%)	11 (33.33%)	
Unknown	6				
**FLT3-ITD**					0.1459
Unmutated	164	22 (78.57%)	122 (73.94%)	20 (58.82%)	
Mutated	63	6 (21.43%)	43 (26.06%)	14 (41.18%)	
**C/EBPα**					0.8575
Unmutated	205	26 (92.86%)	148 (89.70%)	31 (91.18%)	
Mutated	22	2 (7.14%)	17 (10.30%)	3 (8.82%)	
**NPM1**					**0.0471**
Unmutated	169	19 (67.86%)	119 (72.12%)	31 (91.18%)	
Mutated	58	9 (32.14%)	46 (27.88%)	3 (8.82%)	
**Allogeneic HSCT**					0.0548
Untransplanted	147	23 (82.14%)	106 (64.24%)	18 (52.94%)	
Allogeneic transplantation	80	5 (17.86%)	59 (35.76%)	16 (47.06%)	

Supplemental Table 4: TriAS independently predicts RFS of AML patients independently of other established risk factors. Multivariate Cox regression analysis for RFS of AML patients from the NTUH validation set 1 is shown. For other data sets, no relapse data was available.

**NTUH Validation-Set [n=177]**	**HR multivariate**	**P-value multivariate**
**Cytogenetic risk group poor**	**1.929 (1.055-3.528)**	**0.0330**
**Cytogenetic risk group favorable**	**0.337 (0.174-0.655)**	**0.0013**
**C/EBPα mutated**	**0.365 (0.157-0.851)**	**0.0196**
FLT3 mutated	1.396 (0.844-2.309)	0.1940
NPM1 mutated	0.875 (0.498-1.538)	0.6430
Gender (female)	0.840 (0.543-1.300)	0.4344
Age>65	1.209 (0.626-2.335)	0.5715
**TriAS**	**1.334 (1.027-1.732)**	**0.0306**

Supplemental Table 5: Multivariate Cox regression analysis for OS of the CN-AML subcohort from the TCGA training and the NTUH validation set 1 comparing different expression based prediction scores separately

TCGA Training set CN-AML [n=82]	HR multivariate	P-value multivariate
Gender (female)	1.713 (0.988-2.973)	0.0554
**Age >65**	**3.832 (2.073-7.082)**	**<0.0001**
**TriAS**	**2.082 (1.464-2.961)**	**<0.0001**

TCGA Training set CN-AML [n=82]	HR multivariate	P-value multivariate
Gender (female)	1.607 (0.928-2.784)	0.0907
**Age >65**	**2.334 (1.314-4.145)**	**0.0038**
Marcucci score	1.331 (0.756-2.343)	0.3211

TCGA Training set CN-AML [n=82]	HR multivariate	P-value multivariate
Gender (female)	1.537 (0.894-2.643)	0.1198
**Age >65**	**2.448 (1.349-4.443)**	**0.0032**
Chuang score	1.004 (0.959-1.050)	0.8757

TCGA Training set CN-AML [n=82]	HR multivariate	P-value multivariate
Gender (female)	1.669 (0.962-2.894)	0.0683
**Age >65**	**2.255 (1.278-3.977)**	**0.0050**
Li score	1.693 (0.962-2.979)	0.0677

NTUH Validation-Set CN-AML [n=111]	HR multivariate	P-value multivariate
Gender (female)	1.124 (0.657-1.925)	0.6693
**Age >65**	**3.099 (1.647 – 5.832)**	**0.0005**
**TriAS**	**1.560 (1.110 – 2.192)**	**0.0105**

NTUH Validation-Set CN-AML [n=111]	HR multivariate	P-value multivariate
Gender (female)	1.123 (0.654 – 1.927)	0.6749
**Age >65**	**2.615 (1.392 – 4.913)**	**0.0028**
Marcucci score	1.528 (0.894 – 2.612)	0.1211

**NTUH Validation-Set CN-AML [n=111] Parameter**	**HR multivariate**	**P-value multivariate**
Gender (female)	0.887 (0.514 – 1.530)	0.6669
**Age >65**	**2.166 (1.156 – 4.057)**	**0.0158**
**Chuang score**	**1.104 (1.061 – 1.149)**	**<0.0001**

**NTUH Validation-Set CN-AML [n=111] Parameter**	**HR multivariate**	**P-value multivariate**
Gender (female)	1.023 (0.593 – 1.763)	0.9353
**Age >65**	**2.543 (1.357 – 4.764)**	**0.0036**
Li score	1.758 (0.998 – 3.095)	0.0508

Supplemental Table 6: Multivariate Cox regression analysis for OS of the non-CN-AML subcohort from the TCGA training and the NTUH validation set 1 comparing different expression based prediction scores separately

**TCGA Training set non-CN-AML [n=66]**	**HR multivariate**	**P-value multivariate**
Gender (female)	0.887 (0.433-1.816)	0.7434
**Age >65**	**4.318 (1.960-9.514)**	**0.0003**
Cytogenetic risk group poor	0.501 (0.159-1.579)	0.2379
**Cytogenetic risk group favorable**	**0.194 (0.058-0.651)**	**0.0079**
**TriAS**	**2.207 (1.382-3.524)**	**0.0009**

**TCGA Training set non-CN-AML [n=66]**	**HR multivariate**	**P-value multivariate**
Gender (female)	0.723 (0.364-1.438)	0.3553
**Age >65**	**4.934 (2.242-10.858)**	**<0.0001**
Cytogenetic risk group poor	0.551 (0.172-1.760)	0.3143
**Cytogenetic risk group favorable**	**0.230 (0.070-0.756)**	**0.0155**
Marcucci score	1.263 (0.536-2.979)	0.5930

**TCGA Training set non-CN-AML [n=66]**	**HR multivariate**	**P-value multivariate**
Gender (female)	0.701 (0.353-1.390)	0.3088
**Age >65**	**5.190 (2.386-11.289)**	**<0.0001**
Cytogenetic risk group poor	0.602 (0.193-1.877)	0.3820
**Cytogenetic risk group favorable**	**0.235 (0.072-0.773)**	**0.0171**
Chuang score	1.000 (0.934-1.071)	0.9914

**TCGA Training set non-CN-AML [n=66]**	**HR multivariate**	**P-value multivariate**
Gender (female)	0.693 (0.350-1.374)	0.2941
**Age >65**	**5.299 (2.458-11.424)**	**<0.0001**
Cytogenetic risk group poor	0.685 (0.211-2.229)	0.5300
**Cytogenetic risk group favorable**	**0.196 (0.054-0.712)**	**0.0133**
Li score	0.698 (0.257-1.896)	0.4803

**NTUH Validation-Set non-CN-AML [n=110]**	**HR multivariate**	**P-value multivariate**
Gender (female)	0.732 (0.424 – 1.263)	0.2623
Age >65	1.892 (0.831 – 4.305)	0.1286
Cytogenetic risk group poor	2.013 (0.936 – 4.325)	0.0732

Cytogenetic risk group favorable	0.440 (0.187 – 1.037)	0.0607
**TriAS**	**1.389 (1.021 – 1.891)**	**0.0367**

NTUH Validation-Set non-CN-AML [n=110]	HR multivariate	P-value multivariate
Gender (female)	0.816 (0.474 - 1.405)	0.4637
Age >65	2.019 (0.893 - 4.565)	0.0912
**Cytogenetic risk group poor**	**2.216 (1.037 - 4.734)**	**0.0400**
Cytogenetic risk group favorable	0.445 (0.191 - 1.033)	0.0595
**Marcucci score**	**2.361 (1.340 - 4.159)**	**0.0029**

NTUH Validation-Set non-CN-AML [n=110]	HR multivariate	P-value multivariate
Gender (female)	0.890 (0.513 - 1.543)	0.6771
Age >65	1.922 (0.846 - 4.364)	0.1184
**Cytogenetic risk group poor**	**2.559 (1.175 - 5.574)**	**0.0180**
Cytogenetic risk group favorable	0.465 (0.197 - 1.094)	0.0795
**Chuang score**	**1.069 (1.016 - 1.124)**	**0.0105**

NTUH Validation-Set non-CN-AML [n=110]	HR multivariate	P-value multivariate
Gender (female)	0.743 (0.431 - 1.282)	0.2862
Age >65	1.810 (0.799 - 4.102)	0.1551
**Cytogenetic risk group poor**	**2.237 (1.039 - 4.816)**	**0.0396**
Cytogenetic risk group favorable	0.526 (0.219 - 1.264)	0.1511
**Li score**	**1.946 (1.099 - 3.446)**	**0.0224**
